# A novel panel of differentially-expressed microRNAs in breast cancer brain metastasis may predict patient survival

**DOI:** 10.1038/s41598-019-55084-z

**Published:** 2019-12-06

**Authors:** Athina Giannoudis, Kim Clarke, Rasheed Zakaria, Damir Varešlija, Mosavar Farahani, Lucille Rainbow, Angela Platt-Higgins, Stuart Ruthven, Katherine A. Brougham, Philip S. Rudland, Michael D. Jenkinson, Leonie S. Young, Francesco Falciani, Carlo Palmieri

**Affiliations:** 1 Institute of Translational Medicine, Molecular and Clinical Cancer Medicine, University of Liverpool, Liverpool, UK; 2 Computational Biology Facility, University of Liverpool, Liverpool, UK; 3 Institute of Integrative Biology, University of Liverpool, Liverpool, UK; 4 Department of Neurosurgery, The Walton Centre NHS Foundation Trust, Liverpool, UK; 5 Endocrine Oncology Research Group, Department of Surgery, Royal College of Surgeons in Ireland, Dublin, Ireland; 6 Department of Pathology, Royal Liverpool University Hospital, Liverpool, UK; 7 Institute of Translational Medicine, Molecular and Clinical Pharmacology, University of Liverpool, Liverpool, UK; 8 The Clatterbridge Cancer Centre NHS Foundation Trust, Wirral, UK

**Keywords:** Breast cancer, miRNAs

## Abstract

Breast cancer brain metastasis (BCBM) is an area of unmet clinical need. MicroRNAs (miRNAs) have been linked to the metastatic process in breast cancer (BC). In this study, we aim to determine differentially-expressed miRNAs utilising primary BCs that did not relapse (BCNR, n = 12), primaries that relapsed (BCR) and their paired (n = 40 pairs) brain metastases (BM) using the NanoString™ nCounter™ miRNA Expression Assays. Significance analysis of microarrays identified 58 and 11 differentially-expressed miRNAs between BCNR vs BCR and BCR vs BM respectively and pathway analysis revealed enrichment for genes involved in invasion and metastasis. Four miRNAs, miR-132-3p, miR-199a-5p, miR-150-5p and miR-155-5p, were differentially-expressed within both cohorts (BCNR-BCR, BCR-BM) and receiver-operating characteristic curve analysis (p = 0.00137) and Kaplan-Meier survival method (p = 0.0029, brain metastasis-free survival; p = 0.0007, overall survival) demonstrated their potential use as prognostic markers. Ingenuity pathway enrichment linked them to the MET oncogene, and the cMET protein was overexpressed in the BCR (p < 0.0001) and BM (p = 0.0008) cases, compared to the BCNRs. The 4-miRNAs panel identified in this study could be potentially used to distinguish BC patients with an increased risk of developing BCBM and provide potential novel therapeutic targets, whereas cMET-targeting warrants further investigation in the treatment of BCBM.

## Introduction

Despite the improvements in the treatment of breast cancer (BC), recurrence and metastasis remain a major clinical problem. Breast cancer brain metastases (BCBM) are a growing clinical problem, and an area of unmet clinical need^1,2^. During the invasive-metastatic cascade, malignant cells must develop stem-cell characteristics, evade the immune system and adapt to the different microenvironments through which they pass in order to metastasise and seed in the target organ^3,4^. MicroRNAs (miRNAs), may interact with numerous mRNAs and differentially modulate their expression and the corresponding protein levels in a tissue and cell type-specific manner^5^. In the context of cancer, miRNAs are known to regulate a number of disease-related protein-coding genes, having therefore, diagnostic and prognostic potential, as well as being potential therapeutic targets^3,5–9^.

Several studies using BC cell lines, mouse models and primary or advanced BC clinical samples have identified miRNAs linked to the metastatic process^3,5–10^. For instance, higher levels of miRNA-10b in the primary BC correlated with concurrent BCBM, with *in-vitro* and *in-vivo* data demonstrating that miRNA-10b promotes invasion by inhibition of HOXD10, which in turn de-represses the expression of the pro-metastatic RhoC gene^6,8,10,11^. MiRNA-21 is another BC-related miRNA that promotes *in-vitro* and *in-vivo* tumour growth by targeting PTEN and TPM1 tumour-suppressor genes, whereas it affects metastasis by directly targeting TIMP3, SERPIN5B, PDCD4 and BCL2^8,9^.

To date, only three studies with very limited numbers of samples (n = 4–9), have compared miRNA expression between matched primary unselected BC and BCBM tissues^12–14^. The current study screened a larger cohort of unselected BC samples that recur (BCR) and their paired brain metastases (BM), as well as primary BC samples that did not recur (BCNR) to identify differentially-expressed miRNAs related to BCBM. Such information is vital if biomarkers or therapeutic targets are to be identified which could transform the prevention and treatment of BCBM.

## Results

### Patient characteristics

A total of 12 BCNR and 40 BCR patients who developed BM were included in the study. Among the primary BCNR cohort, 9 (75%) were ER+ and 3 (25%) were HER2+, whereas in the BCR cohort, 21 (52.5%) were ER+, 11 (27.5%) were HER2+ and 8 (20%) were triple negative (TNs). The distribution of the subtypes changed in the BM setting with the majority of ER+ primary BCRs losing their ER status; 10/21 (47.6%) ER+ and 3/5 (60%) HER2+/ER+ lost ER expression. The data is summarised in Table 1.

**Table 1:** Hormone receptor (ER and PgR) and HER2 status of the primary BCR and their matched BCBM cases.

Patient N^o^	Status	ER status of the 1^o^BC	ER status of BM	PgR status of the 1^o^BC	PgR status of BM	HER2 status of the 1^o^BC	HER2 status of BM	Time (months) between breast-brain surgeries/recurrence	Time (months) between breast surgery and death
119	ER+	+	+	−	+	−	−	62	85
560	ER+/PgR+	+	+	+	+	−	−	97	103
666	ER+/PgR+	+	−	+	−	−	−	38	55
690	ER+/PgR+	+	+	+	+	−	−	44	72
707	HER2+/ER+	+	−	−	−	+	+	48	89
712	ER+	+	−	−	−	−	−	28	32
725	ER+/PgR+	+	−	+	+	−	−	22	26
756	HER2+/ER+	+	−	−	−	+	+*	25	53
827	TN	−	−	−	+	−	−	16	NA
912	ER+/PgR+	+	+	+	+	−	−	60	94
943	TN	−	−	−	−	−	−	36	61
972	HER2+/HR+	+	−	+	+	+	+	27	29
1004	ER+	+	−	−	+	−	−	10	NA
1020	TN	−	−	−	+	−	−	26	NA
1148	ER+/PgR+	+	+	+	+	−	−	51	52
1386	ER+	+	−	−	−	−	−	11	NA
1662	ER+	+	−	−	+	−	−	39	54
1709	TN	−	−	−	+	−	−	23	25
12051	ER+	+	−	−	−	−	−	6	29
12321	ER+	+	−	−	−	−	−	14	64
12372	HER2+	−	NA	−	NA	+	NA	14	25
12364	HER2+	−	−	−	−	+	+	29	64
13147	ER+/PgR+	+	−	+	−	−	−	24	42
13263	ER+	+	+	−	−	−	−	30	42
13630	TN	−	−	−	−	−	−	30	31
13631	HER2+	−	−	−	NA	+	+*	21	30
13692	TN	−	−	−	NA	−	−	35	44
14063	TN	−	−	−	NA	−	−	26	31
14222	ER+/PgR+	+	+	+	+	−	−	Synchronous	50
15311	ER+/PgR+	+	+	+	+	−	−	NA	174
RCSI_1	HER2+	−	−	−	−	+	+	20	31
RCSI_2	HER2+/ER+	+	+	−	−	+	+	37	104
RCSI_3	HER2+	−	−	−	−	+	+	67	115
RCSI_5	ER+	+	+	−	−	−	+	53	74
RCSI_6	TN	−	−	−	−	−	−	23	40
RCSI_7	ER+	+	+	−	−	−	+	53	73
RCSI_8	ER+	+	+	−	−	−	−	59	97
RCSI_9	HER2+	−	−	−	−	+	+	17	40
RCSI_10	HER2+	−	−	−	−	+	+	70	112
RCSI_11	ER+	+	−	−	−	−	−	8	17

The table indicates the differences of the ER, PgR and HER2 status between the primary BCR and BCBM cases. Samples were collected from Walton Tissue Bank, Liverpool, UK and Royal College of Surgeons Ireland (RCSI) National Breast Cancer Bioresource, Ireland.

NA: Not available,+: positive, −: negative.

*2+, FISH not available.

### Differentially expressed miRNAs between primary BCNR vs BCR and BCR vs BCBMs

**Figure 1:**
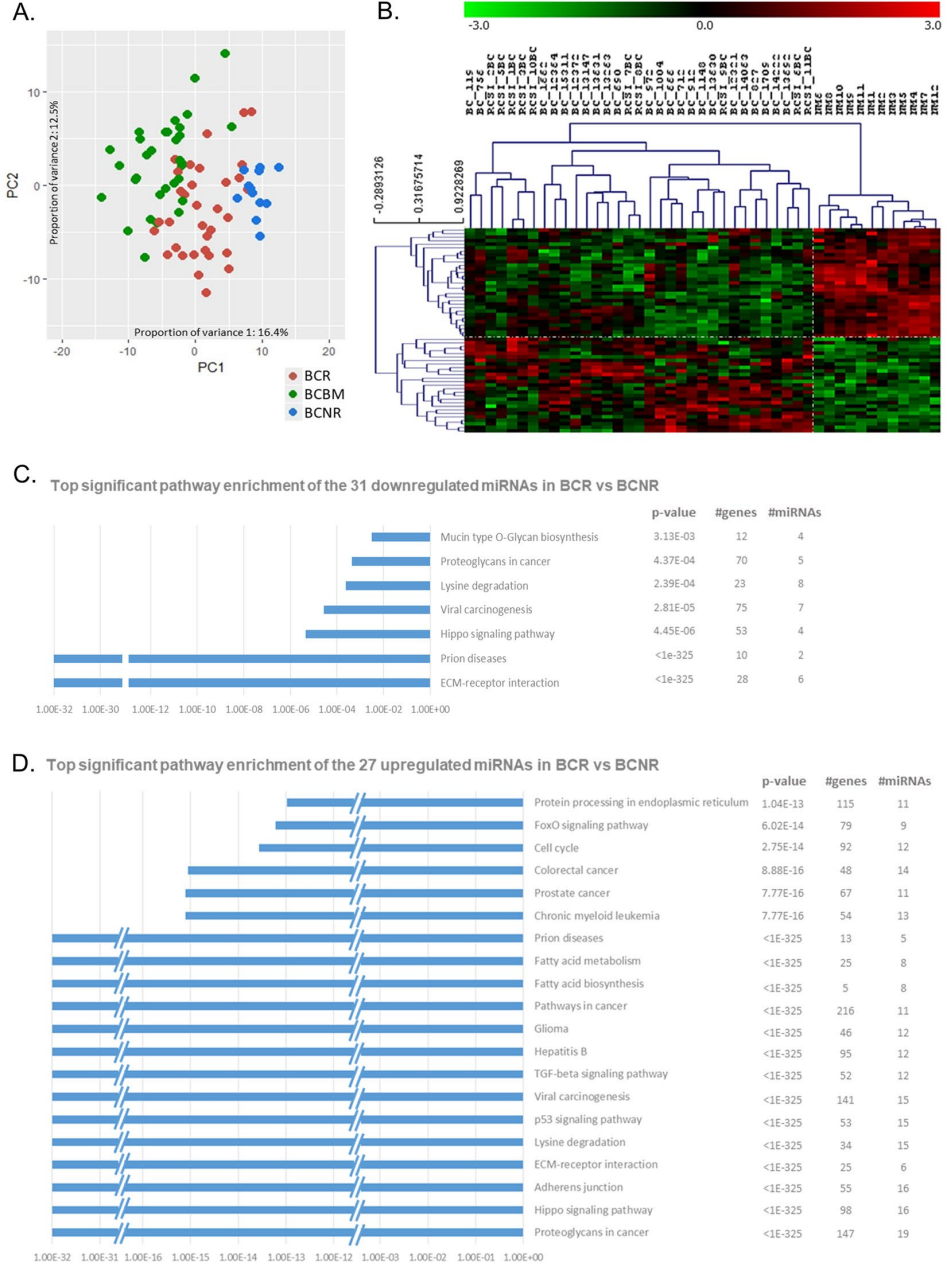
Principal component analysis and miRNA expression profiling of primary breast cancer (BC) without recurrence and primary BC that recurs to the brain. (**A**) Principal component analysis (PCA) of the miRNA expression values after sample and array normalisation. PC1 (horizontal axis) accounted for 16.1%, while PC2 (vertical axis) accounted for 12.5% of the observed variation. The blue spots represented the primary BCs that did not recur (BCNR), whereas the red and green spots represented the primary BC that recurred in the brain (BCR) and BCBM samples respectively. There is an observed shift from the BCNR to BCR and from BCR to BCBM highlighting the molecular distinction. (**B**) Significant analysis of microarrays (SAM) with 10% false discovery rate (FDR) and fold-change (FC) > 2 separates the primary BCNR (NM in the dendrogram) from the primary BCRs (BC and RSCI_BC in the dendrogram). 31 miRNAs were downregulated (green) and 27 were upregulated (red) in BCRs in comparison to the BCNRs. Pathway enrichment analysis (DIANA-mirPath v3.0) of the (**C**) 31 downregulated and (**D**) 27 upregulated miRNAs in BCRs in comparison to the BCNRs with the top significant KEGG pathways plotted.

Assessment of the global variation using principal component analysis (PCA) applied to the miRNA counts indicated a molecular distinction between the primary BCNR (blue dots), the BCRs (red dots) that progress to brain metastasis and the BCBM (green dots) cases, with a small number of overlapping samples mainly between BCRs and BCBMs (Fig. 1A).

Significance analysis of microarrays (SAM) identified 116 differentially-expressed miRNAs between primary BCNRs and BCRs with 10% false discovery rate (FDR) (Supplementary Table 1), 58 of which (31 downregulated and 27 upregulated miRNAs in BCR versus BCNR) had absolute log_2_ fold-changes (FC) > 1 (Fig. 1B, Supplementary Table 1: dark and light-grey respectively). Pathway clustering (DIANA-mirPath_v3.0) revealed enrichment for genes involved in extracellular matrix (ECM)-receptor interactions, proteoglycans, p53, TGF-β, hippo and FOXO signalling, fatty acid biosynthesis/degradation/metabolism, adherens junctions and cell cycle (Fig. 1C,D, Supplementary Fig. 1A,B). Core ingenuity pathway analysis (IPA) identified a network of genes implicated in proliferation of immune cells, migration and metastasis, directly and/or indirectly regulated by the identified miRNA signature (Supplementary Fig. 2A–D).

**Figure 2:**
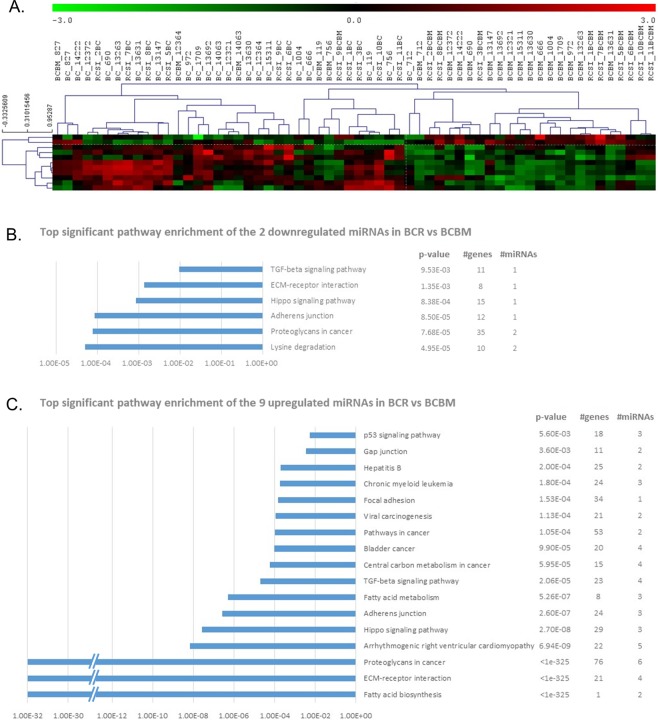
miRNA expression profiling and pathway enrichment analysis of the primary breast cancer (BCR) and their paired breast cancer brain metastasis (BCBM). (**A**) Significant analysis of microarrays (SAM) with 10% false discovery rate (FDR) and fold-change (FC) > 2 distinguishes the primary BCR (BC, RSCI_BC in the dendrogram) from their paired BCBM (BCBM, RSCI_BCBM in the dendrogram). 2 miRNAs were downregulated (green) and 9 were upregulated (red) in BCRs in comparison to the BCBMs. Pathway enrichment analysis (DIANA-mirPath v3.0) of the (**B**) 2 downregulated and (**C**) 9 upregulated miRNAs in BCRs in comparison to the BCNRs with the top significant KEGG pathways plotted.

Comparison of the primary BCRs and their paired BMs by SAM revealed 112 differentially-expressed miRNAs with 10% FDR (Supplementary Table 2), 11 of which (9 upregulated and 2 downregulated in BCR versus their paired BMs) had log_2_ FC > 1 (Fig. 2A, Supplementary Table 2: light and dark-grey respectively). The 11 miRNAs are highly enriched for genes involved in ECM-receptor interactions, proteoglycans, adherens junctions, p53, TGF-β, hippo signalling, fatty acid biosynthesis/degradation/metabolism and focal adhesion (DIANA-mirPath_v3.0) (Fig. 2B,C, Supplementary Fig. 3A,B). IPA core analysis identified a network of genes implicated in proliferation of immune cells, metastasis and colonisation, directly and/or indirectly regulated by the identified miRNA signature (Supplementary Fig. 4A–D).

### A signature of miRNAs may define distant metastasis to the brain

**Figure 3:**
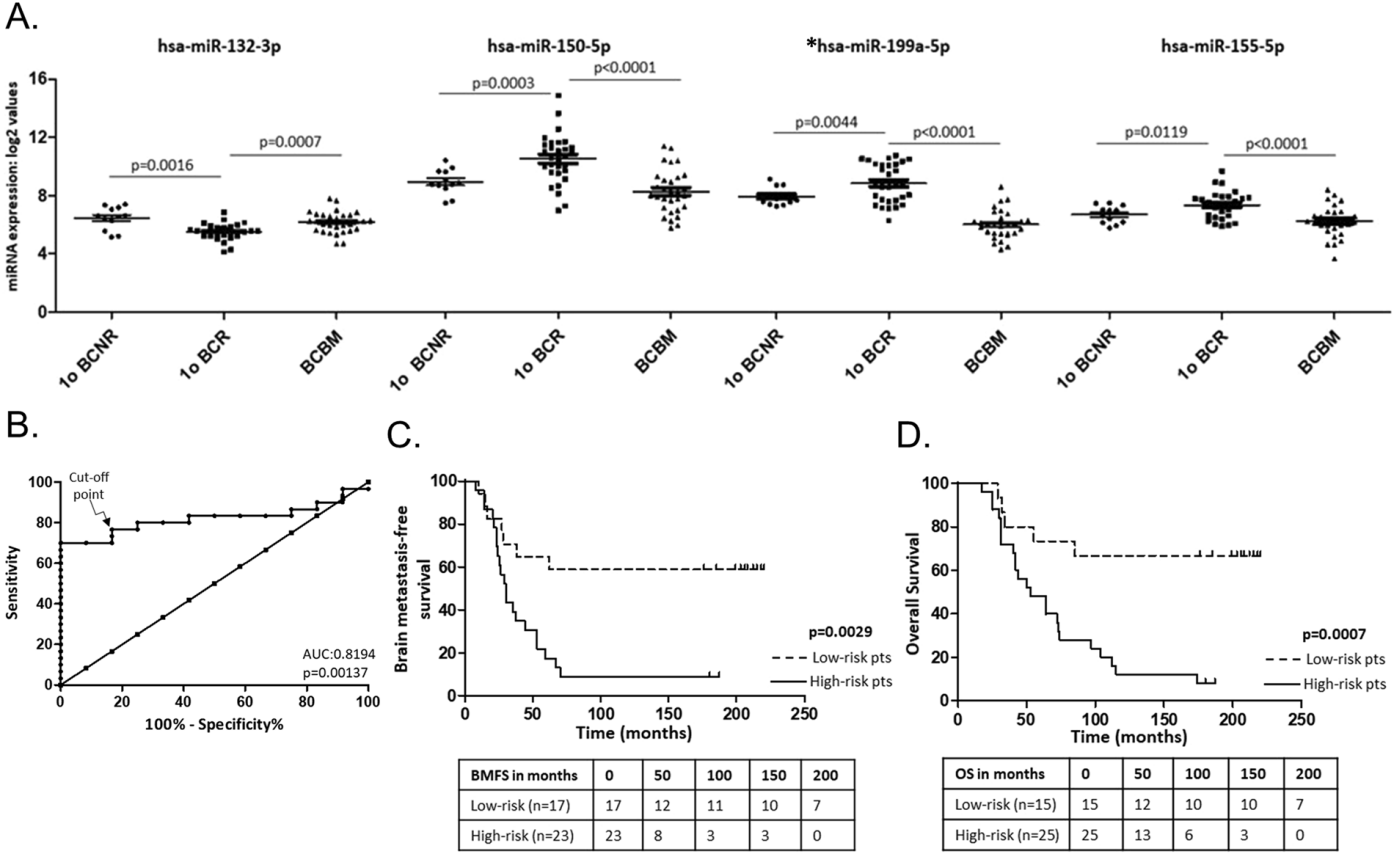
Prognostic potential of the 4-miRNA signature. (**A)** T-test (Welch’s correction) was used to compare the primary BCNR with BCRs and Wilcoxon signed-rank test (Gaussian approximation) was used to compare the BCR and their paired BCBMs. *Only hsa-miR-199a-5p showed a significant difference (p < 0.0001) in expression between BCNR and BCBM. **(B)** Receiver-operating characteristic (ROC) curve analyses of the mean log_2_ expression of the four-miRNA signature was used to discriminate BCNR (control group) from BCR (metastasis group). The identified area under the curve (AUC = 0.8194) supports the prognostic potential of this 4-miRNA signature. (**C**,**D**) Kaplan-Meier survival analysis showed that low-risk patients had a better brain metastasis-free survival (**C**) and overall survival (**D**) than the high-risk with p = 0.0029 and p = 0.0007 respectively.

Four of the significantly differentially-expressed miRNAs identified by SAM, hsa-miR-132-3p, hsa-miR-199a-5p, hsa-miR-150-5p and hsa-miR-155-5p were present within both cohorts of BCNR vs BCR and BCR vs BCBM (Fig. 3A). Statistical analysis (t-test) on the individual miRNAs showed a p < 0.01 and p < 0.0001 for all these miRNAs between BCNR vs BCR and BCR vs BCBM respectively (Fig. 3A). Only the hsa-miR-199a-5p showed a significant p value (p < 0.0001) between BCNR and BCBM. The differential expression of the 4 miRNAs within BCNR vs BCR (irrespectively of BC subtype) and BCR vs BCBM cohorts leads us to focus and investigate them further. In order to discriminate BCNR from BCR and study the diagnostic accuracy of these miRNAs as biomarkers for brain metastasis, receiver-operating characteristic curve (ROC) analysis was performed (Fig. 3B) using their mean log_2_-expression values. The area under the curve (AUC) was 0.8194 (95% CI: 0.6932–0.9457, p = 0.00137), meaning that a patient (BCR) will have a more abnormal test result than 81.94% of the controls (BCNR). The cut-off level of the combined 4-miRNA was 7.892 with 76.7% sensitivity and 83.3% specificity. This cut-off was used to dichotomised the primary BC cases into high-risk (>7.892) and low-risk (<7.892) and was used to perform Kaplan-Meier (log-rank) analysis of BMFS and OS. The result showed that low-risk group had a higher BMFS rate (p = 0.0029, HR: 0.314, 95% CI: 0.146–0.673) and a better overall survival (p = 0.0007, HR: 0.267, 95% CI: 0.124–0.573) than the high-risk group (Fig. 3C,D respectively), confirming that the expression levels of the 4-miRNA signature were significantly correlated with patient outcome. The individual miRNA contribution in BMFS and OS is illustrated in Supplementary Fig. 5. High miR-132-3p expression confers a protective effect whereas high miR-150-5p, miR-155-5p and miR-199a-5p confer a risky effect (HR < 1 and HR > 1 respectively).

**Figure 4:**
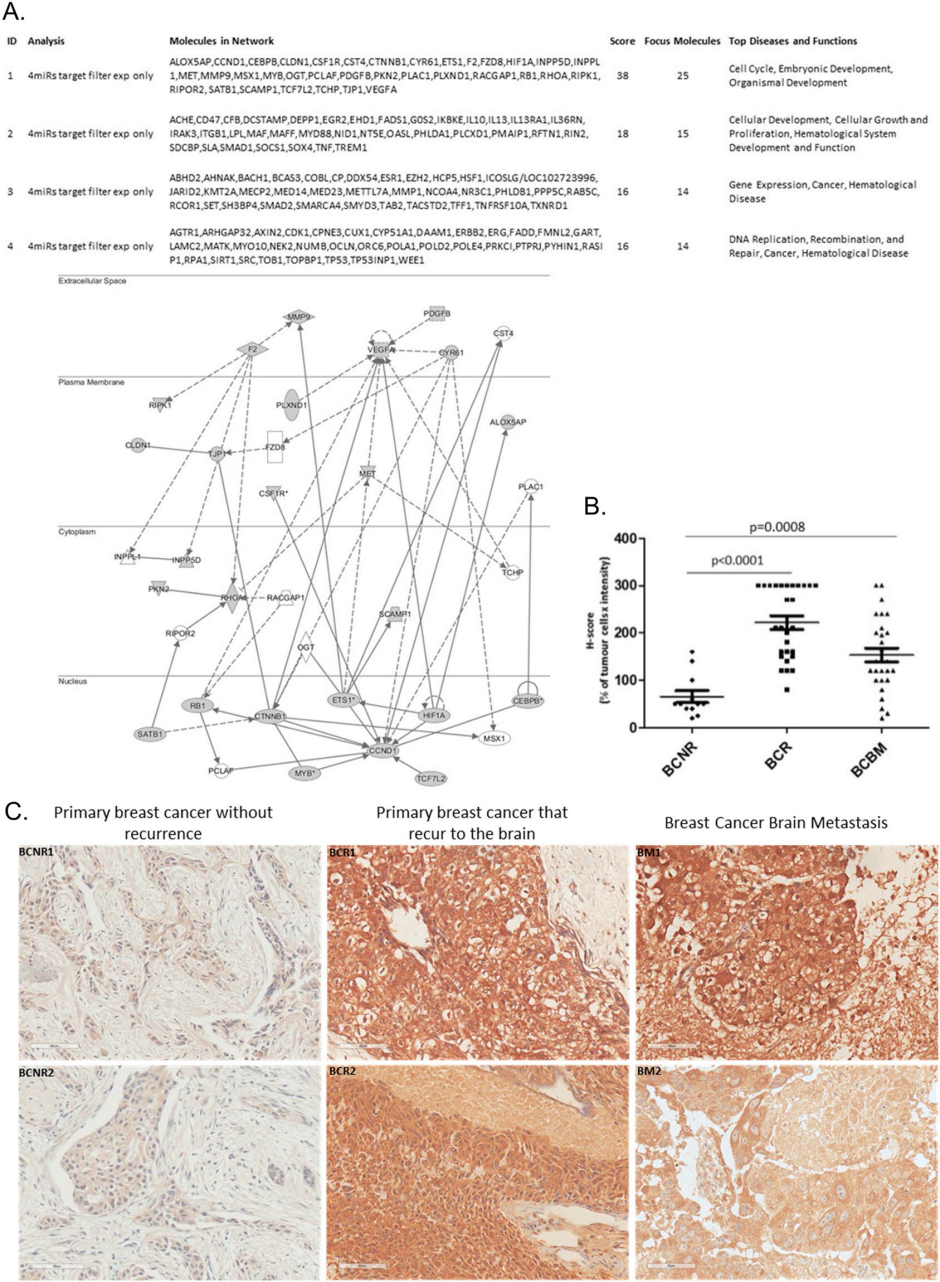
Ingenuity pathway network analysis of the genes targeted by the 4 differentially-expressed miRNAs and cMET immunohistochemistry of primary breast cancer that did not recur (BCNR), primary BCR and breast cancer brain metastasis (BCBM). (**A)** IPA of the 147 genes targeted enrichment analysis linked them to 4 major networks (ID1-4), the top identified network (ID 1) is illustrated with the highest score and focus molecules. MET (cMET) oncogene was one of the genes in this network **(B**) cMET IHC using the H-score as a semi-quantitative measure for the t-test analysis identified a significant overexpression of cMET in BCRs and in BCBMs (p < 0.0001: BCNR vs BCR and p = 0.0008: BCNR vs BCBM). (**C**) Representative examples stained for cMET of 2 primary breast cancer cases without recurrence (BCNR1 and BCNR2) and 2 primary BCR with their matched brain metastasis (BCR1 and BM1, BCR2 and BM2).

IPA’s miRNA target-filter functionality with filtering for experimentally validated targets identified 147 genes linked to 4 major networks (Fig. 4A). MET (cMET, HGF human growth factor ligand) oncogene was one of the genes in the top identified network (ID1, Fig. 4A) and it was also present in the IPA core analysis of the differentially-expressed miRNAs between BCNR vs BCR (Supplementary Fig. 2A) and BCR vs BCBM (Supplementary Fig. 4A). The cMET was overexpressed in primary BCRs and their paired BMs in comparison to the primary BCNRs (Fig. 4B: BCNR vs BCR: p < 0.0001 and BCNR vs BCBM: p = 0.0008), indicating an activation of the HGF/cMET pathway in the BCs that relapse and in BM. Representative examples of cMET staining of two primary breast cancer cases without recurrence (BCNR) and 2 primary BCRs with their matched brain metastasis are illustrated in Fig. 4C.

## Discussion

In this study, we investigated the differential-expression of miRNAs between primary BCNR vs primary BCR and primary BCR vs their paired BMs. Assessment of the global variation using PCA indicated a molecular distinction between the primary BCNR, the BCRs that progress to brain metastasis and the BCBM cases. The overlap observed between the primary BCRs and their paired BMs suggests that many of the miRNAs linked to metastasis are already present in a proportion of the matched primary breast tumours. Consistent with the literature, we observed hormone (ER and/or PgR) receptor loss in BCBM cases^15–17^. Since brain metastasis is a late event in hormone receptor-positive BC, cases losing hormone sensitivity may behave more like TNs that are known to have a higher rate of BM and a worse prognosis.

Comparison of the primary BC samples that do not relapse with the ones that relapse, revealed 58 differentially-expressed miRNAs, many of which have been previously shown to be involved in breast tumourigenesis, progression and metastasis targeting a number of key oncogenes or tumour-suppressor genes^6–10,14,18,19^. Inflammation and immunity have been also shown to contribute to the development and progression of BC and a number of the identified miRNAs including miR-150, miR-155, miR-146, miR-130/301 and the miR-17/92 cluster are involved in regulating genes and pathways in these processes^3,20–27^. IPA linked them to interleukins (IL), toll-like receptors (TLR) and C-reactive protein (CRP), whereas miRNA target-filter analysis linked them to ILs, TLR, Th1/Th2 and neuro-inflammation signalling pathways; (Supplementary Fig. 6A) highlighting the activation of an inflammatory network involved in the metastatic process.

To identify miRNAs that may be associated with BCBM, we analysed a cohort of primary BCs that recur and their paired BMs. This revealed a signature of 11 differentially-expressed miRNAs with previously published *in-vitro* and *in-vivo* data supporting their relevance in the processes of invasion and metastasis^8–12,14,24,28,29^. For instance, higher expression of miR-155-5p has been reported in localised early breast cancer than in metastasis, including brain metastasis^28^. The miR-155-5p promotes cancer cell extravasation by regulating the function of the blood-brain endothelial barrier and its overexpression shifts the TGF-β response from growth inhibition to EMT transition, invasion and metastasis in breast cancer through loss of C/EBP-β^3,8,23,24^. Moreover, miR-199a-3p and miR-214-3p, both up-regulated in human BC stem cells, enhance stem cell behaviour and facilitate metastasis by targeting Ezh2, p53, PTEN, BIM, and Snail^8,26,30^. Within our current cohort of primary BCs and their paired BMs, miR-10b-5p, miR-150-5p were upregulated while, miR-132-3p, and miR-9-5p were downregulated. These miRNAs have been linked to the induction of mesenchymal markers early in the EMT transition and to neovascularisation, both facilitating metastatic growth^3,8–10,31^. Overexpression of miR-150 promotes growth, clonogenicity and reduces apoptosis in breast cancer cells^29,32^, whereas primary BCs with increased levels of miRNA-10b have been associated with lymph node metastasis, *de novo* metastatic disease, as well as brain metastasis^3,6,8–11^. Consistent with our data, miR-9 has been reported to be upregulated in non-CNS metastatic disease when compared to the primary BC, supporting its role in metastasis and colonisation^14^. In comparison to normal tissue, miR-132-3p is downregulated in primary BC, whereas its overexpression in cell lines and animal models, significantly suppress BC cell proliferation, invasion, migration and metastasis^33–35^. Interestingly, miR-132-3p has been characterised as a ‘neuro-immunomiR’, a class of miRNAs which operate within and between the neural and immune compartments supporting its potential importance in brain metastasis^36^. A recent study showed that over-expression of miR-212/132 in hypoxic mouse and human brain microvascular endothelial cells decreased the blood-brain-barrier properties by targeting the transcription of tight junction and tight junction-associated proteins, highlighting further the importance of this miRNA in the brain^37^. MiR-132, miR-155, miR-150 and miR-10b regulate both innate and adaptive immunity and in the context of cancer, their immunomodulatory effects can enable malignant cells to evade the immune system^21–23,25,38^. The 11 differentially-expressed miRNAs within the primary BCR and their paired BMs were also linked by IPA to genes and pathways involved in inflammation and immunity, cell stemness and EMT transition. IPA target-filter analysis linked them to ILs, PI3K and neuroinflammation signalling pathways (Supplementary Fig. 6B), highlighting further the activation of an inflammatory network. The current data demonstrate the potential importance of immuno-regulation in the biology of BCBM and the potential for the use of immuno-oncology agents in their treatment^39,40^.

Four of the differentially-expressed miRNAs within the primary BCRs and their paired BMs, miR-132-3p, miR-150-5p, miR-199a-5p, miR-155-5p, were also differentially-expressed in the cohort of primary BCNRs as compared to primary BCRs. This observation lead us to focus and investigate them further. To evaluate their prognostic significance, we calculated the AUC of the ROC curve for development of brain metastasis using the BCNR patients as non-metastatic controls. The high AUC value indicated that the 4-miRNAs can distinguish a patient that will relapse to the brain from the one who will not, supporting the prognostic potential of the identified 4-miRNAs signature. Further to the ROC curve analysis, Kaplan-Meier survival plot showed that the low-risk group had a 60% brain metastasis-free survival rate and 67% OS, whereas the high-risk group had metastasis-free and overall survival rate lower than 10%.

Of interest, the oncogene MET, encoding the transmembrane receptor cMET, was common in all the IPA network analyses. In BC, cMET is associated with aggressive clinico-pathological features, shorter disease-free/overall survival and brain metastasis^41–43^. Consistent with these data, we observed cMET overexpression in BCR and their paired BMs but not in the BCNR samples. Previously, we also demonstrated increased cMET copy number in paired primary BCR and their BMs^44^. The above, highlight an active cMET pathway in primary BC samples that recurred to the brain as well as in BCBM but not in primary BC cases that do not recur.

Microglial cells are known to express cMET and produce HGF, contributing to angiogenesis of brain tumours^42,45,46^. A paracrine cytokine loop present between tumour-associated astrocytes and cancer cells results in continuous activation of the cMET pathway leading to induction of angiogenesis^42^. Therefore, cMET appears able to promote brain colonisation and maintenance via promoting angiogenesis within the CNS. Silencing cMET in combination with radiotherapy in a brain metastatic mouse model significantly prolonged survival, demonstrating its therapeutic potential^47^. cMET activation is also know to modulate immune cell functions, therefore, inhibition of cMET signalling may not only suppress cancer cell growth but also stimulate the immune system by alleviating the immunosuppressive effects on macrophages and dendritic cells^45,46,48^. Given that there are available drugs targeting cMET, the potential benefit of targeting cMET-positive BCBM with these agents warrants investigation.

A limitation of our study is the challenge in collecting a larger number of matched primary BC and BM cases. Moreover, it had been previously observed that the miRNA levels gradually decreased over time in long-archived FFPE blocks^49^ and this accounted for the limited number of BCNR cases with a long follow-up available in our study. Therefore, an analysis by subtypes was not feasible. In an attempt to reduce the possible effect of the TN cases (absent in our BCNR cohort) we further analysed the BCNR vs ER + /HER2 + BCR (Supplementary Table 3). Of the 4 miRNAs, miR-132-3p, miR-150-5p, miR-199a-5p and miR-155-5p, only miR-155-5p was absent in the combined BCNR vs ER + /HER2 + BCR analysis indicating that its expression may be more relevant to TNBC^23,28^. However, given the hormone loss in the metastatic setting and the previous report of miR-155-5p in brain metastasis, we believe that this miRNA is still important and its role requires further investigation. In addition, the majority of patients with metastatic breast cancer, might present metastases to other organs such as bones, liver, lungs prior to the development of BM. However, the presence of BM regardless of other metastatic sites is a critical clinical complication, conferring a dismal prognosis to the patients. The patients included in this study came from the biobank of a specialist tertiary referral neurosurgical centre (Walton Hospital) and the relevant detailed clinical information with regard to other involved anatomical sites was not collected by the biobank. Given the samples are anonymised we were unable to seek out and collect this information. Finally, the patients included in this study may have received some form of systemic therapy and/or radiotherapy prior to the biopsy of the metastatic lesion. Therefore, the detected miRNAs might be affected by the metastatic process, the therapy or a combination of both. Despite these limitations, this study provides a comprehensive analysis of differentially-expressed miRNAs in primary BCs that did not relapsed versus primary BCs that relapsed to the brain and their matched brain metastasis. It should be also noted, that the current study represents the largest set of unselected matched primary breast tumours and brain metastases so far.

In summary, this is the first study to report a panel of differentially-expressed miRNAs in BCBM that could potentially be used to identify BC patients at increased risk of BCBM as well as provide potential novel therapeutic targets. Since these miRNAs were also linked to cMET using network analysis, the data supports a novel therapeutic strategy for BCBM that is focused on targeting cMET.

## Methods

### Patients

A total of 12 formalin-fixed paraffin-embedded (FFPE) primary BCNR and 40 primary BCR samples with their paired BMs were collected from the Liverpool Tissue Bank, Walton Research Tissue Bank (WRTB) Liverpool, UK and the Royal College of Surgeons Ireland (RCSI) National Breast Cancer Bioresource, Ireland. The specimens were reviewed by two pathologists (SR,KB) and defined as per local protocol. Briefly, ER, PgR positivity was defined using the Allred scoring system (3–8), whereas HER2 status was classified as negative (staining 0–1), positive (staining 3 + ). Amplification of HER2 was confirmed by FISH (fluorescent *in-situ* hybridisation) if the staining was 2 + (equivocal). The cMET protein expression was assessed by immunohistochemistry (IHC). Intensity was scored according to a four-tier systems: 0, no staining; 1+, weak; 2+, moderate; and 3+, strong. The H-score was used as a semi-quantitative measure by multiplying the staining intensity (0–3) and the percentage of positive cells (0–100%) for a final IHC score ranging from 0 to 300. The study was performed in accordance with the Declaration of Helsinki and approved by the WRTB Ethics committee (WRTB15_06), the National Research Ethics Committee (NRES 11/WN003/2), the UK Health Research Authority (NRES 12/NW/0778) and the RCSI Institutional Review Board (#13/09; ICORG09/07). Appropriate approvals and written consent were in place before anonymised tissue and data were released.

### RNA extraction and miRNA profiling

RNA was extracted using the miRNeasy FFPE kit (Qiagen, Manchester, UK) and quantified on the ND-Nanodrop1000 spectrometer (ThermoFisher Scientific, Wilmington, MA, USA). RNA integrity number (RIN) was determined using the 2100 Agilent Bioanalyzer (Agilent Technologies, Palo Alto, CA). Profiling was performed using the NanoString™ nCounter™ miRNA Expression Assay (Human_v3 miRNA) according to the manufacturer’s instructions. The raw data were quality control (QC) assessed and normalised by the NanoString™ nSolver™ analysis software. Fifteen cases (5 paired BCR-BM, 2 BCRs and 3 BMs) failed the NanoString™ QC and normalisation and were excluded from downstream analysis together with their matched samples. MiRNAs with median expression lower than the upper quartile of the in-built negative controls were removed and an additional quantile normalisation procedure (R-Bioconductor) was applied to remove technical variation between different arrays resulting in a final dataset containing 166 miRNAs (Supplementary File 1). Principle component analysis (PCA) of the normalised log_2_-transformed data was done using the prcomp function within the R software for statistical analysis. Significance analysis of microarrays (SAM) was performed on the Multi-Experiment viewer (MeV) 4.9 software to identify differentially-expressed miRNAs between BCNR vs BCR and BCR vs their paired BM^50^.

### Pathway analysis

Pathway analysis was carried out using DIANA-mirPath_v3.0 a web-server, utilizing the DIANA-microT-CDS algorithm, the DIANA-TarBase and the Kyoto Encyclopedia of Genes and Genomes (KEGG) pathways^51^. Fisher’s Exact test (hypergeometric distribution) corrected for multiple testing with p < 0.05 threshold was used to define pathway enrichment. Ingenuity Pathway Analysis (IPA, Qiagen bioinformatics) was used for network and miRNA gene-target enrichment analysis.

### Statistical analysis

Two sample unpaired t-test (Welch’s correction) was used to compare the primary BCNR with the BCRs and Wilcoxon signed-rank paired t-test (Gaussian approximation) was used to compare the BCR and their paired BMs. Receiver-operating characteristic (ROC) curve analysis of the mean log_2_ expression of the four-miRNAs signature was used to discriminate BCNR (control) from BCR (cases) patients and evaluate the diagnostic accuracy of the selected miRNAs. The median patient follow-up of the BCNR controls was 17 years. Kaplan-Meier (Log-rank) survival analysis was used to determine the brain metastasis-free survival (BMFS: time between breast-brain surgeries/recurrence) and overall survival (OS: time between breast surgery to death). All the statistical analysis was performed on GraphPad Prism v5 software (GraphPad Inc, San Diego, USA).

### Ethics approval and consent to participate

This project was peer reviewed by the Walton Research Tissue Bank (WRTB15_06), an approved repository under the Human Tissue Act (National Research Ethics Service: NRES 11/WN003/2), the UK Health Research Authority (NRES 12/NW/0778) and the RCSI Institutional Review Board (#13/09; ICORG09/07). Appropriate approvals and written consent were in place before anonymised tissue and data were released. The study was performed in accordance with the Declaration of Helsinki.

## Supplementary information

SREP-19-21066A_Supplementary Info

Additional file 1

## Data Availability

All the data (raw counts and normalised counts) analysed in this study are available in the Additional File 1 (Additional file 1_Raw Counts and Normalised Data.xlsx) submitted with the manuscript.
