# Supplementary material for: A novel panel of differentially-expressed microRNAs in breast cancer brain metastasis may predict patient survival
Source: Sci Rep. 2019 Dec 6;9:18518. doi: 10.1038/s41598-019-55084-z (PMC6897960; doi:10.1038/s41598-019-55084-z)

### A novel panel of differentially-expressed microRNAs in breast cancer brain metastasis may predict patient survival

Athina Giannoudis^1^, Kim Clarke^2^, Rasheed Zakaria^3,4^, Damir Varešlija^5^, Mosavar Farahani^1^, Lucille Rainbow^3^, Angela Platt-Higgins^3^, Stuart Ruthven^6^, Katherine A Brougham^6^, Philip S Rudland^3^, Michael D Jenkinson^4,7^, Leonie S Young^5^, Francesco Falciani^2^, *Carlo Palmieri^1,8^.

1. Institute of Translational Medicine, Molecular and Clinical Cancer Medicine, University of Liverpool, Liverpool, UK
2. Computational Biology Facility, University of Liverpool, Liverpool, UK
3. Institute of Integrative Biology, University of Liverpool, Liverpool, UK
4. Department of Neurosurgery, The Walton Centre NHS Foundation Trust, Liverpool, UK
5. Endocrine Oncology Research Group, Department of Surgery, Royal College of Surgeons in Ireland, Dublin, Ireland
6. Department of Pathology, Royal Liverpool University Hospital, Liverpool, UK
7. Institute of Translational Medicine, Molecular and Clinical Pharmacology, University of Liverpool, Liverpool, UK
8. The Clatterbridge Cancer Centre NHS Foundation Trust, Wirral, UK

***Correspondence to:**

Professor Carlo Palmieri,

University of Liverpool,

Institute of Translational Medicine,

Molecular and Clinical Cancer Medicine,

Sherrington Building,

Ashton Street,

Liverpool, L69 3GE, UK.

Tel.: +44 (0)151 794 9815;

Email: [c.palmieri@liverpool.ac.uk](mailto:c.palmieri@liverpool.ac.uk)

**Supplementary Information**

**Supplementary Table 1: Significant Analysis of Microarrays (SAM) on MeV software.** SAM (d) identified 116 miRNAs differentially expressed between primary BCs that did not recur (BCNR) and BCs that recur (BCR) with 10% FDR (q value). The 58 miRNAs with log2 FC>1 (light grey: 27 upregulated in BCR) and log2 FC<-1 (dark grey: 31 downregulated in BCR) compared to BCNRs were used for further analysis. *FC: Fold Change.

| **miRNAS** | **SAM (d)** | **FC (unlogged)** | **q value (%)** | **log2FC** |
| --- | --- | --- | --- | --- |
| hsa-miR-125a-5p | -2.5539 | 0.3093 | 0.0000 | -1.6931 |
| hsa-miR-324-5p | -2.2836 | 0.3138 | 0.0000 | -1.6720 |
| hsa-miR-19b-3p | -2.3000 | 0.3147 | 0.0000 | -1.6679 |
| hsa-miR-93-5p | -2.2309 | 0.3488 | 0.0000 | -1.5194 |
| hsa-miR-25-3p | -2.0584 | 0.3575 | 0.0000 | -1.4838 |
| hsa-miR-151a-5p | -1.8934 | 0.3583 | 0.0000 | -1.4808 |
| hsa-miR-30d-5p | -1.9000 | 0.3680 | 0.0000 | -1.4422 |
| hsa-miR-20a-5p + hsa-miR-20b-5p | -1.7512 | 0.3722 | 0.0000 | -1.4259 |
| hsa-miR-99b-5p | -1.6981 | 0.3780 | 0.0000 | -1.4034 |
| hsa-miR-200b-3p | -1.7051 | 0.3858 | 0.0000 | -1.3741 |
| hsa-miR-125b-5p | -1.6603 | 0.3872 | 0.0000 | -1.3690 |
| hsa-miR-146a-5p | -1.3993 | 0.4003 | 0.5746 | -1.3210 |
| hsa-miR-200c-3p | -1.6990 | 0.4084 | 0.0000 | -1.2918 |
| hsa-miR-130a-3p | -1.6762 | 0.4148 | 0.0000 | -1.2697 |
| hsa-miR-150-5p | -1.4980 | 0.4203 | 0.0000 | -1.2507 |
| hsa-miR-100-5p | -1.6161 | 0.4226 | 0.0000 | -1.2427 |
| hsa-miR-365a-3p + hsa-miR-365b-3p | -1.3530 | 0.4269 | 0.5659 | -1.2281 |
| hsa-miR-19a-3p | -1.3849 | 0.4298 | 0.5746 | -1.2183 |
| hsa-miR-16-5p | -1.8210 | 0.4457 | 0.0000 | -1.1659 |
| hsa-miR-199a-5p | -1.0790 | 0.4458 | 1.4940 | -1.1656 |
| hsa-miR-107 | -1.1479 | 0.4475 | 1.5349 | -1.1600 |
| hsa-miR-21-5p | -1.6736 | 0.4668 | 0.0000 | -1.0992 |
| hsa-miR-181a-5p | -1.4941 | 0.4729 | 0.0000 | -1.0803 |
| hsa-miR-98-5p | -1.2496 | 0.4769 | 0.5413 | -1.0683 |
| hsa-miR-361-3p | -1.6182 | 0.4825 | 0.0000 | -1.0515 |
| hsa-miR-361-5p | -1.3213 | 0.4866 | 0.5493 | -1.0393 |
| hsa-miR-106a-5p + hsa-miR-17-5p | -0.9856 | 0.4875 | 2.2500 | -1.0365 |
| hsa-miR-126-3p | -1.4013 | 0.5088 | 0.5746 | -0.9747 |
| hsa-miR-15b-5p | -1.0481 | 0.5156 | 1.9154 | -0.9557 |
| hsa-miR-221-3p | -0.9619 | 0.5186 | 2.2500 | -0.9472 |
| hsa-miR-223-3p | -1.1771 | 0.5244 | 1.5562 | -0.9314 |
| hsa-miR-148b-3p | -0.9054 | 0.5296 | 2.5758 | -0.9170 |
| hsa-miR-155-5p | -0.8901 | 0.5321 | 2.5465 | -0.9102 |
| hsa-miR-142-3p | -1.0355 | 0.5336 | 1.9154 | -0.9062 |
| hsa-miR-30b-5p | -0.7618 | 0.5470 | 5.1264 | -0.8705 |
| hsa-miR-195-5p | -1.0287 | 0.5483 | 1.9154 | -0.8670 |
| hsa-miR-127-3p | -0.8362 | 0.5499 | 2.8112 | -0.8627 |
| hsa-let-7f-5p | -0.7671 | 0.5507 | 5.1264 | -0.8608 |
| hsa-miR-146b-5p | -0.9105 | 0.5523 | 2.5758 | -0.8564 |
| hsa-miR-99a-5p | -0.8401 | 0.5565 | 2.8112 | -0.8455 |
| hsa-miR-423-5p | -0.6921 | 0.5566 | 5.9965 | -0.8453 |
| hsa-miR-342-3p | -0.9678 | 0.5580 | 2.2500 | -0.8417 |
| hsa-miR-222-3p | -0.9761 | 0.5593 | 2.2500 | -0.8382 |
| hsa-miR-10a-5p | -0.7359 | 0.5631 | 5.4392 | -0.8287 |
| hsa-miR-135b-5p | -0.6953 | 0.5714 | 5.9965 | -0.8074 |
| hsa-miR-374b-5p | -0.8350 | 0.5744 | 2.8112 | -0.7998 |
| hsa-miR-24-3p | -1.5323 | 0.5935 | 0.0000 | -0.7526 |
| hsa-miR-199b-5p | -0.6642 | 0.5978 | 5.9420 | -0.7424 |
| hsa-miR-145-5p | -0.8136 | 0.5988 | 4.9545 | -0.7398 |
| hsa-miR-140-5p | -0.7892 | 0.6001 | 4.9545 | -0.7367 |
| hsa-miR-26a-5p | -0.7470 | 0.6008 | 5.1264 | -0.7349 |
| hsa-miR-374a-5p | -0.8581 | 0.6098 | 2.8112 | -0.7136 |
| hsa-miR-191-5p | -1.1229 | 0.6219 | 1.5142 | -0.6852 |
| hsa-miR-30a-5p | -0.7951 | 0.6292 | 4.9545 | -0.6684 |
| hsa-let-7i-5p | -0.8766 | 0.6304 | 2.5179 | -0.6656 |
| hsa-miR-4286 | -0.7176 | 0.6365 | 5.9965 | -0.6518 |
| hsa-miR-193b-3p | -0.5717 | 0.6437 | 8.1907 | -0.6355 |
| hsa-miR-30c-5p | -0.7074 | 0.6591 | 5.9965 | -0.6013 |
| hsa-miR-10b-5p | -0.5637 | 0.6623 | 8.1907 | -0.5944 |
| hsa-miR-23b-3p | -1.3130 | 0.6643 | 0.5493 | -0.5900 |
| hsa-miR-29a-3p | -0.7491 | 0.6662 | 5.1264 | -0.5860 |
| hsa-miR-27b-3p | -0.9645 | 0.6691 | 2.2500 | -0.5798 |
| hsa-miR-660-5p | -0.7184 | 0.6725 | 5.9965 | -0.5723 |
| hsa-miR-144-3p | -0.6884 | 0.6977 | 5.9965 | -0.5194 |
| hsa-miR-451a | -0.9016 | 0.6992 | 2.5758 | -0.5163 |
| hsa-let-7d-5p | -0.5992 | 0.7002 | 7.0030 | -0.5141 |
| hsa-let-7e-5p | -0.5431 | 0.7016 | 8.6191 | -0.5113 |
| hsa-miR-340-5p | -0.7855 | 0.7277 | 4.9545 | -0.4586 |
| hsa-miR-455-5p | -0.5310 | 0.7332 | 8.6191 | -0.4477 |
| hsa-miR-34a-5p | -0.6395 | 0.7621 | 6.2249 | -0.3919 |
| hsa-miR-425-5p | -0.9090 | 0.7626 | 2.5758 | -0.3910 |
| hsa-miR-186-5p | 1.0228 | 1.2941 | 1.5562 | 0.3719 |
| hsa-miR-205-5p | 1.0152 | 1.4501 | 1.5562 | 0.5362 |
| hsa-miR-28-3p | 0.8576 | 1.4775 | 4.3707 | 0.5632 |
| hsa-miR-590-5p | 1.3238 | 1.6023 | 0.0000 | 0.6801 |
| hsa-miR-183-5p | 1.3355 | 1.6035 | 0.0000 | 0.6812 |
| hsa-miR-539-5p | 1.2990 | 1.6398 | 0.0000 | 0.7135 |
| hsa-miR-185-5p | 1.2117 | 1.6770 | 0.6024 | 0.7459 |
| hsa-miR-363-3p | 1.4475 | 1.6960 | 0.0000 | 0.7621 |
| hsa-miR-493-3p | 1.3845 | 1.7170 | 0.0000 | 0.7799 |
| hsa-miR-1915-3p | 1.5196 | 1.8146 | 0.0000 | 0.8596 |
| hsa-miR-1290 | 1.6590 | 1.8364 | 0.0000 | 0.8769 |
| hsa-miR-181b-5p + hsa-miR-181d-5p | 1.5671 | 1.8697 | 0.0000 | 0.9028 |
| hsa-miR-181a-3p | 1.3622 | 1.9037 | 0.0000 | 0.9288 |
| hsa-miR-551a | 1.7682 | 1.9691 | 0.0000 | 0.9775 |
| hsa-miR-378g | 1.5298 | 2.0288 | 0.0000 | 1.0206 |
| hsa-miR-769-5p | 1.6027 | 2.0412 | 0.0000 | 1.0294 |
| hsa-miR-4521 | 1.8793 | 2.0752 | 0.0000 | 1.0532 |
| hsa-miR-362-5p | 1.6700 | 2.1258 | 0.0000 | 1.0880 |
| hsa-miR-379-5p | 1.9824 | 2.1297 | 0.0000 | 1.0906 |
| hsa-miR-582-5p | 1.8296 | 2.1322 | 0.0000 | 1.0923 |
| hsa-miR-374c-5p | 1.8541 | 2.1817 | 0.0000 | 1.1254 |
| hsa-miR-299-3p | 1.7851 | 2.1978 | 0.0000 | 1.1361 |
| hsa-miR-299-5p | 1.8544 | 2.2543 | 0.0000 | 1.1727 |
| hsa-miR-320e | 1.8903 | 2.3507 | 0.0000 | 1.2331 |
| hsa-miR-337-3p | 2.0073 | 2.4368 | 0.0000 | 1.2850 |
| hsa-miR-337-5p | 2.0605 | 2.4475 | 0.0000 | 1.2913 |
| hsa-miR-3161 | 2.3355 | 2.4810 | 0.0000 | 1.3109 |
| hsa-miR-95-3p | 2.0672 | 2.6379 | 0.0000 | 1.3994 |
| hsa-miR-132-3p | 2.1621 | 2.6861 | 0.0000 | 1.4255 |
| hsa-miR-4488 | 2.3462 | 2.7299 | 0.0000 | 1.4488 |
| hsa-miR-495-3p | 2.5355 | 2.8119 | 0.0000 | 1.4915 |
| hsa-miR-664a-3p | 2.5848 | 2.8220 | 0.0000 | 1.4967 |
| hsa-miR-575 | 2.5238 | 2.8521 | 0.0000 | 1.5120 |
| hsa-miR-487a-3p | 2.6696 | 2.9550 | 0.0000 | 1.5632 |
| hsa-miR-543 | 2.6738 | 2.9759 | 0.0000 | 1.5733 |
| hsa-miR-598-3p | 2.7214 | 3.0499 | 0.0000 | 1.6088 |
| hsa-miR-27a-3p | 2.6686 | 3.1237 | 0.0000 | 1.6433 |
| hsa-miR-4284 | 2.6452 | 3.1514 | 0.0000 | 1.6560 |
| hsa-miR-514b-5p | 2.8567 | 3.1745 | 0.0000 | 1.6665 |
| hsa-miR-630 | 2.7319 | 3.4342 | 0.0000 | 1.7800 |
| hsa-miR-378f | 3.3671 | 3.8646 | 0.0000 | 1.9503 |
| hsa-miR-1973 | 3.1078 | 3.8779 | 0.0000 | 1.9553 |
| hsa-miR-4516 | 3.0472 | 3.8932 | 0.0000 | 1.9610 |
| hsa-miR-574-5p | 3.0575 | 3.9825 | 0.0000 | 1.9937 |
| hsa-miR-610 | 2.3627 | 4.3886 | 0.0000 | 2.1337 |

**Supplementary Table 2: Significant Analysis of Microarrays (SAM) on MeV software.** SAM (d) identified 112 miRNAs differentially expressed between primary BC that recur (BCR) and their paired BC brain metastasis (BCBM) with 10% FDR (q value). The 11 miRNAs with log2 FC>1 (light grey: 9 upregulated in BCR) and log2 FC<-1 (dark grey: 2 downregulated in BCR) were used for further analysis. *FC: Fold Change.

| **miRNA names** | **SAM (d)** | **FC (unlogged)** | **q value (%)** | **log2FC** |
| --- | --- | --- | --- | --- |
| hsa-miR-199a-5p | -3.5775 | 0.3020 | 0.0000 | -1.7275 |
| hsa-miR-199b-5p | -3.5039 | 0.3172 | 0.0000 | -1.6567 |
| hsa-miR-199a-3p + hsa-miR-199b-3p | -3.5570 | 0.3510 | 0.0000 | -1.5104 |
| hsa-miR-214-3p | -2.9546 | 0.3852 | 0.0000 | -1.3764 |
| hsa-miR-150-5p | -2.5040 | 0.3984 | 0.0000 | -1.3279 |
| hsa-miR-10b-5p | -3.2221 | 0.4197 | 0.0000 | -1.2527 |
| hsa-miR-145-5p | -2.1167 | 0.4424 | 0.0000 | -1.1765 |
| hsa-miR-222-3p | -2.4718 | 0.4643 | 0.0000 | -1.1069 |
| hsa-miR-155-5p | -2.3673 | 0.4979 | 0.0000 | -1.0061 |
| hsa-miR-146b-5p | -2.1434 | 0.5422 | 0.0000 | -0.8832 |
| hsa-miR-125b-5p | -1.4282 | 0.5502 | 0.0000 | -0.8618 |
| hsa-miR-142-3p | -2.0100 | 0.5554 | 0.0000 | -0.8485 |
| hsa-miR-22-3p | -2.1256 | 0.5593 | 0.0000 | -0.8382 |
| hsa-miR-4286 | -1.3853 | 0.5660 | 0.0000 | -0.8212 |
| hsa-miR-126-3p | -1.4647 | 0.5798 | 0.0000 | -0.7864 |
| hsa-let-7i-5p | -1.6052 | 0.5828 | 0.0000 | -0.7789 |
| hsa-miR-424-5p | -1.9383 | 0.5897 | 0.0000 | -0.7620 |
| hsa-miR-497-5p | -1.7473 | 0.6262 | 0.0000 | -0.6754 |
| hsa-miR-99a-5p | -1.5265 | 0.6409 | 0.0000 | -0.6417 |
| hsa-miR-337-5p | -1.3244 | 0.6557 | 0.0000 | -0.6090 |
| hsa-miR-378i | -0.9462 | 0.6699 | 1.5882 | -0.5780 |
| hsa-miR-376a-3p | -1.2635 | 0.6885 | 0.0000 | -0.5384 |
| hsa-miR-23b-3p | -1.2730 | 0.6910 | 0.0000 | -0.5332 |
| hsa-miR-195-5p | -1.7388 | 0.6936 | 0.0000 | -0.5279 |
| hsa-miR-4488 | -1.0381 | 0.6970 | 1.0127 | -0.5208 |
| hsa-miR-130a-3p | -1.5620 | 0.7019 | 0.0000 | -0.5107 |
| hsa-miR-205-5p | -1.4229 | 0.7099 | 0.0000 | -0.4944 |
| hsa-miR-10a-5p | -1.9170 | 0.7130 | 0.0000 | -0.4881 |
| hsa-miR-136-5p | -0.9943 | 0.7221 | 1.4165 | -0.4698 |
| hsa-miR-378g | -0.9314 | 0.7249 | 1.9902 | -0.4642 |
| hsa-miR-100-5p | -0.7525 | 0.7431 | 4.1327 | -0.4285 |
| hsa-miR-337-3p | -0.9482 | 0.7519 | 1.5882 | -0.4114 |
| hsa-miR-1915-3p | -0.8991 | 0.7695 | 2.1305 | -0.3780 |
| hsa-let-7c-5p | -1.2644 | 0.7712 | 0.0000 | -0.3749 |
| hsa-miR-146a-5p | -1.1875 | 0.7837 | 0.0000 | -0.3517 |
| hsa-miR-23a-3p | -1.2373 | 0.8093 | 0.0000 | -0.3052 |
| hsa-miR-514b-5p | -0.7536 | 0.8143 | 4.1327 | -0.2963 |
| hsa-miR-26a-5p | -0.8216 | 0.8167 | 3.5332 | -0.2920 |
| hsa-miR-26b-5p | -0.8550 | 0.8176 | 2.5258 | -0.2904 |
| hsa-miR-30e-5p | -0.5967 | 0.8308 | 9.7580 | -0.2674 |
| hsa-miR-335-5p | -0.7783 | 0.8384 | 3.8395 | -0.2543 |
| hsa-miR-455-5p | -0.8203 | 0.8425 | 3.5332 | -0.2472 |
| hsa-miR-127-3p | -0.6686 | 0.8438 | 7.0917 | -0.2451 |
| hsa-miR-28-3p | -0.7759 | 0.8642 | 3.8395 | -0.2105 |
| hsa-miR-450a-5p | -1.0457 | 0.8677 | 1.0127 | -0.2047 |
| hsa-miR-503-5p | -1.2196 | 0.8919 | 0.0000 | -0.1651 |
| hsa-miR-382-5p | -0.6186 | 0.9018 | 9.3820 | -0.1491 |
| hsa-let-7g-5p | -0.7378 | 0.9020 | 4.0887 | -0.1488 |
| hsa-miR-1290 | 0.5452 | 0.9973 | 8.0630 | -0.0039 |
| hsa-miR-539-5p | 0.5168 | 1.0517 | 9.3820 | 0.0727 |
| hsa-miR-1260a | 0.8760 | 1.0938 | 1.9902 | 0.1294 |
| hsa-miR-664a-3p | 0.4755 | 1.1229 | 10.2948 | 0.1672 |
| hsa-miR-30a-5p | 0.5176 | 1.1264 | 9.3820 | 0.1717 |
| hsa-miR-141-3p | 0.6043 | 1.1355 | 6.6386 | 0.1833 |
| hsa-miR-324-5p | 0.8526 | 1.1402 | 2.1568 | 0.1893 |
| hsa-miR-374c-5p | 0.5050 | 1.1426 | 9.4562 | 0.1923 |
| hsa-miR-331-3p | 0.9571 | 1.1606 | 0.9842 | 0.2149 |
| hsa-miR-4284 | 0.4916 | 1.1857 | 9.3702 | 0.2457 |
| hsa-miR-590-5p | 0.8584 | 1.1920 | 2.1568 | 0.2534 |
| hsa-miR-135b-5p | 0.5371 | 1.1933 | 8.3190 | 0.2550 |
| hsa-miR-200b-3p | 1.2011 | 1.2045 | 0.0000 | 0.2684 |
| hsa-miR-3161 | 0.6144 | 1.2074 | 6.6386 | 0.2720 |
| hsa-miR-106b-5p | 0.9842 | 1.2272 | 0.9983 | 0.2954 |
| hsa-miR-362-5p | 0.6107 | 1.2479 | 6.6386 | 0.3195 |
| hsa-miR-181b-5p + hsa-miR-181d-5p | 1.1411 | 1.2878 | 0.5635 | 0.3649 |
| hsa-miR-16-5p | 1.1923 | 1.2983 | 0.0000 | 0.3766 |
| hsa-miR-98-5p | 0.9044 | 1.3083 | 1.3976 | 0.3877 |
| hsa-miR-25-3p | 0.9264 | 1.3094 | 0.9573 | 0.3889 |
| hsa-let-7a-5p | 0.5611 | 1.3115 | 7.6325 | 0.3913 |
| hsa-miR-451a | 0.7647 | 1.3157 | 3.2502 | 0.3958 |
| hsa-miR-106a-5p + hsa-miR-17-5p | 0.7757 | 1.3195 | 3.2884 | 0.4000 |
| hsa-miR-454-3p | 1.2428 | 1.3224 | 0.0000 | 0.4032 |
| hsa-miR-196a-5p | 0.5920 | 1.3305 | 7.1935 | 0.4119 |
| hsa-miR-181a-5p | 0.6789 | 1.3347 | 5.0429 | 0.4166 |
| hsa-let-7f-5p | 0.6906 | 1.3352 | 5.0429 | 0.4170 |
| hsa-miR-423-3p | 0.9984 | 1.3389 | 1.0127 | 0.4211 |
| hsa-miR-1180-3p | 1.6517 | 1.3584 | 0.0000 | 0.4419 |
| hsa-miR-374b-5p | 0.7767 | 1.3669 | 3.2884 | 0.4509 |
| hsa-miR-200c-3p | 1.6574 | 1.3802 | 0.0000 | 0.4648 |
| hsa-miR-125a-5p | 1.3006 | 1.3810 | 0.0000 | 0.4657 |
| hsa-miR-144-3p | 0.6787 | 1.3914 | 5.0429 | 0.4766 |
| hsa-miR-99b-5p | 1.2824 | 1.4034 | 0.0000 | 0.4889 |
| hsa-miR-1246 | 1.2610 | 1.4098 | 0.0000 | 0.4954 |
| hsa-miR-543 | 0.7542 | 1.4186 | 3.4137 | 0.5045 |
| hsa-miR-185-5p | 1.1380 | 1.4266 | 0.5635 | 0.5126 |
| hsa-miR-210-3p | 1.1556 | 1.4331 | 0.5635 | 0.5191 |
| hsa-miR-200a-3p | 1.2613 | 1.4573 | 0.0000 | 0.5433 |
| hsa-miR-148b-3p | 1.0505 | 1.4718 | 0.5546 | 0.5576 |
| hsa-miR-598-3p | 1.0203 | 1.4726 | 1.0127 | 0.5584 |
| hsa-miR-30c-5p | 1.3079 | 1.4768 | 0.0000 | 0.5625 |
| hsa-miR-7-5p | 1.3769 | 1.4778 | 0.0000 | 0.5634 |
| hsa-miR-769-5p | 1.1708 | 1.5005 | 0.0000 | 0.5854 |
| hsa-miR-194-5p | 1.0059 | 1.5099 | 1.0127 | 0.5944 |
| hsa-miR-301a-3p | 1.2941 | 1.5263 | 0.0000 | 0.6100 |
| hsa-miR-149-5p | 1.4579 | 1.5264 | 0.0000 | 0.6101 |
| hsa-miR-151a-5p | 1.5848 | 1.5363 | 0.0000 | 0.6194 |
| hsa-miR-151a-3p | 1.4150 | 1.5678 | 0.0000 | 0.6487 |
| hsa-miR-93-5p | 1.8285 | 1.5763 | 0.0000 | 0.6565 |
| hsa-miR-183-5p | 1.5003 | 1.5847 | 0.0000 | 0.6642 |
| hsa-miR-630 | 0.9950 | 1.5946 | 1.0127 | 0.6732 |
| hsa-miR-340-5p | 0.9323 | 1.6090 | 0.9573 | 0.6861 |
| hsa-miR-30d-5p | 1.3774 | 1.6222 | 0.0000 | 0.6979 |
| hsa-miR-30b-5p | 1.8041 | 1.6274 | 0.0000 | 0.7025 |
| hsa-miR-495-3p | 1.1697 | 1.6505 | 0.0000 | 0.7229 |
| hsa-miR-191-5p | 1.5074 | 1.6747 | 0.0000 | 0.7439 |
| hsa-miR-429 | 1.9332 | 1.7191 | 0.0000 | 0.7816 |
| hsa-miR-363-3p | 1.8258 | 1.7278 | 0.0000 | 0.7890 |
| hsa-miR-15b-5p | 2.0609 | 1.8406 | 0.0000 | 0.8802 |
| hsa-miR-425-5p | 1.9925 | 1.8632 | 0.0000 | 0.8978 |
| hsa-miR-107 | 2.2987 | 1.9985 | 0.0000 | 0.9989 |
| hsa-miR-132-3p | 2.1994 | 2.2621 | 0.0000 | 1.1776 |
| hsa-miR-9-5p | 3.0054 | 2.2659 | 0.0000 | 1.1801 |

**Supplementary Table 3: Significant Analysis of Microarrays (SAM) on MeV software.** SAM (d) identified 82 miRNAs differentially expressed between primary ER+/HER2+ BC that did not recur (BCNR) and primary ER+/HER2+ BC that recur (BCR) with 10% FDR. The 3 miRNAs, miR-132-3p, miR-150-5p and miR-199a-5p highlighted in red are also differentially expressed between BCNR vs BCR (Supplementary table 1) and BCR vs BCBM (Supplementary table 2). *FC: Fold Change.

| **ER+/HER2+ BCR vs ER+/HER2+ BCNR** | **SAM (d)** | **FC (Unlogged)** | **Log2 FC** |
| --- | --- | --- | --- |
| **miR_name** |  |  |  |
| hsa-miR-378f | -5.5500 | 0.1351 | -2.8876 |
| hsa-miR-610 | -3.2534 | 0.1674 | -2.5787 |
| hsa-miR-4516 | -4.6186 | 0.1901 | -2.3952 |
| hsa-miR-574-5p | -4.7545 | 0.1974 | -2.3409 |
| hsa-miR-630 | -4.1321 | 0.2082 | -2.2643 |
| hsa-miR-1973 | -4.8019 | 0.2180 | -2.1978 |
| hsa-miR-514b-5p | -3.8945 | 0.2757 | -1.8589 |
| hsa-miR-543 | -3.6317 | 0.2766 | -1.8540 |
| hsa-miR-598-3p | -3.7870 | 0.2819 | -1.8270 |
| hsa-miR-4284 | -3.7000 | 0.3166 | -1.6590 |
| hsa-miR-95-3p | -3.6204 | 0.3180 | -1.6531 |
| hsa-miR-487a-3p | -3.4822 | 0.3266 | -1.6143 |
| hsa-miR-575 | -3.5345 | 0.3440 | -1.5396 |
| hsa-miR-495-3p | -3.5335 | 0.3467 | -1.5284 |
| hsa-miR-27a-3p | -3.0112 | 0.3865 | -1.3713 |
| hsa-miR-299-5p | -2.3182 | 0.4190 | -1.2548 |
| hsa-miR-582-5p | -2.5901 | 0.4221 | -1.2444 |
| hsa-miR-337-3p | -2.4134 | 0.4249 | -1.2349 |
| hsa-miR-362-5p | -2.7596 | 0.4356 | -1.1988 |
| hsa-miR-378g | -2.5728 | 0.4501 | -1.1518 |
| hsa-miR-337-5p | -2.4132 | 0.4525 | -1.1441 |
| hsa-miR-320e | -2.3916 | 0.4745 | -1.0755 |
| hsa-miR-664a-3p | -2.9211 | 0.4904 | -1.0279 |
| hsa-miR-4488 | -3.1329 | 0.5091 | -0.9740 |
| hsa-miR-132-3p | -2.1890 | 0.5234 | -0.9339 |
| hsa-miR-4521 | -2.2788 | 0.5325 | -0.9092 |
| hsa-miR-3161 | -2.5389 | 0.5509 | -0.8601 |
| hsa-miR-299-3p | -1.9355 | 0.5549 | -0.8498 |
| hsa-miR-181a-3p | -1.7705 | 0.5591 | -0.8389 |
| hsa-miR-363-3p | -1.8401 | 0.5811 | -0.7832 |
| hsa-miR-374c-5p | -2.0433 | 0.5858 | -0.7716 |
| hsa-miR-769-5p | -1.9342 | 0.5865 | -0.7698 |
| hsa-miR-379-5p | -2.1626 | 0.5873 | -0.7679 |
| hsa-miR-551a | -1.9762 | 0.5927 | -0.7546 |
| hsa-miR-183-5p | -1.7724 | 0.5928 | -0.7544 |
| hsa-miR-181b-5p+hsa-miR-181d-5p | -1.8236 | 0.6252 | -0.6777 |
| hsa-miR-590-5p | -1.7603 | 0.6560 | -0.6083 |
| hsa-miR-1915-3p | -1.8610 | 0.6586 | -0.6026 |
| hsa-miR-205-5p | -1.5442 | 0.6845 | -0.5470 |
| hsa-miR-493-3p | -1.5994 | 0.6908 | -0.5336 |
| hsa-miR-185-5p | -1.3107 | 0.7122 | -0.4896 |
| hsa-miR-1290 | -2.1227 | 0.7153 | -0.4834 |
| hsa-miR-539-5p | -1.4169 | 0.7273 | -0.4593 |
| hsa-miR-23b-3p | 1.4185 | 1.4141 | 0.4999 |
| hsa-miR-15b-5p | 1.3095 | 1.4492 | 0.5353 |
| hsa-miR-107 | 1.3729 | 1.5897 | 0.6687 |
| hsa-miR-223-3p | 1.2792 | 1.5968 | 0.6752 |
| hsa-miR-425-5p | 1.6508 | 1.6121 | 0.6890 |
| hsa-miR-361-3p | 1.9646 | 1.6226 | 0.6983 |
| hsa-miR-26a-5p | 1.2989 | 1.6256 | 0.7010 |
| hsa-miR-30b-5p | 1.2637 | 1.6394 | 0.7132 |
| hsa-miR-195-5p | 1.2534 | 1.6436 | 0.7169 |
| hsa-miR-181a-5p | 1.8724 | 1.6593 | 0.7305 |
| hsa-miR-191-5p | 1.5820 | 1.7549 | 0.8114 |
| hsa-miR-24-3p | 2.0089 | 1.7578 | 0.8138 |
| hsa-miR-126-3p | 1.9856 | 1.7759 | 0.8286 |
| hsa-miR-361-5p | 1.6727 | 1.7865 | 0.8371 |
| hsa-miR-19a-3p | 1.4050 | 1.8513 | 0.8886 |
| hsa-miR-151a-5p | 2.2568 | 1.9472 | 0.9614 |
| hsa-miR-130a-3p | 1.7839 | 2.0075 | 1.0054 |
| hsa-miR-423-5p | 1.2882 | 2.1626 | 1.1128 |
| hsa-miR-30d-5p | 2.3463 | 2.3106 | 1.2083 |
| hsa-miR-25-3p | 2.4953 | 2.3350 | 1.2234 |
| hsa-miR-93-5p | 2.7676 | 2.3575 | 1.2373 |
| hsa-miR-100-5p | 2.0939 | 2.3610 | 1.2394 |
| hsa-miR-21-5p | 2.3869 | 2.3710 | 1.2455 |
| hsa-miR-10a-5p | 1.4380 | 2.4078 | 1.2677 |
| hsa-miR-16-5p | 2.6985 | 2.4370 | 1.2851 |
| hsa-miR-146a-5p | 1.7614 | 2.4552 | 1.2958 |
| hsa-miR-324-5p | 3.0496 | 2.5393 | 1.3444 |
| hsa-miR-200b-3p | 2.1704 | 2.6311 | 1.3957 |
| hsa-miR-342-3p | 1.8820 | 2.6532 | 1.4077 |
| hsa-miR-99b-5p | 2.6501 | 2.6756 | 1.4199 |
| hsa-miR-145-5p | 1.7123 | 2.7511 | 1.4600 |
| hsa-miR-199a-5p | 1.8147 | 2.8368 | 1.5043 |
| hsa-miR-200c-3p | 2.2921 | 2.8468 | 1.5093 |
| hsa-miR-20a-5p+hsa-miR-20b-5p | 2.1230 | 3.0066 | 1.5881 |
| hsa-miR-125b-5p | 2.8477 | 3.0800 | 1.6229 |
| hsa-miR-19b-3p | 2.9119 | 3.1087 | 1.6363 |
| hsa-miR-365a-3p+hsa-miR-365b-3p | 2.4712 | 3.3406 | 1.7401 |
| hsa-miR-125a-5p | 4.0302 | 4.1043 | 2.0371 |
| hsa-miR-150-5p | 2.0437 | 4.7992 | 2.2628 |

**Supplementary Figure 1: miRNA pathway enrichment analysis of primary breast cancer (BC) without recurrence (BCNR) and primary BC that recurs to the brain (BCR).** Pathway Union heatmap of the **(A)** 31 downregulated and **(B)** 27 upregulated miRNAs in BCRs in comparison to the BCNRs (dark and light grey respectively in supplementary table 2). The top significant KEGG pathways are indicated. The significance of enrichment is indicated by the strength of the colour (log_10_ p value ranging from 0 to 10^-15^).

**
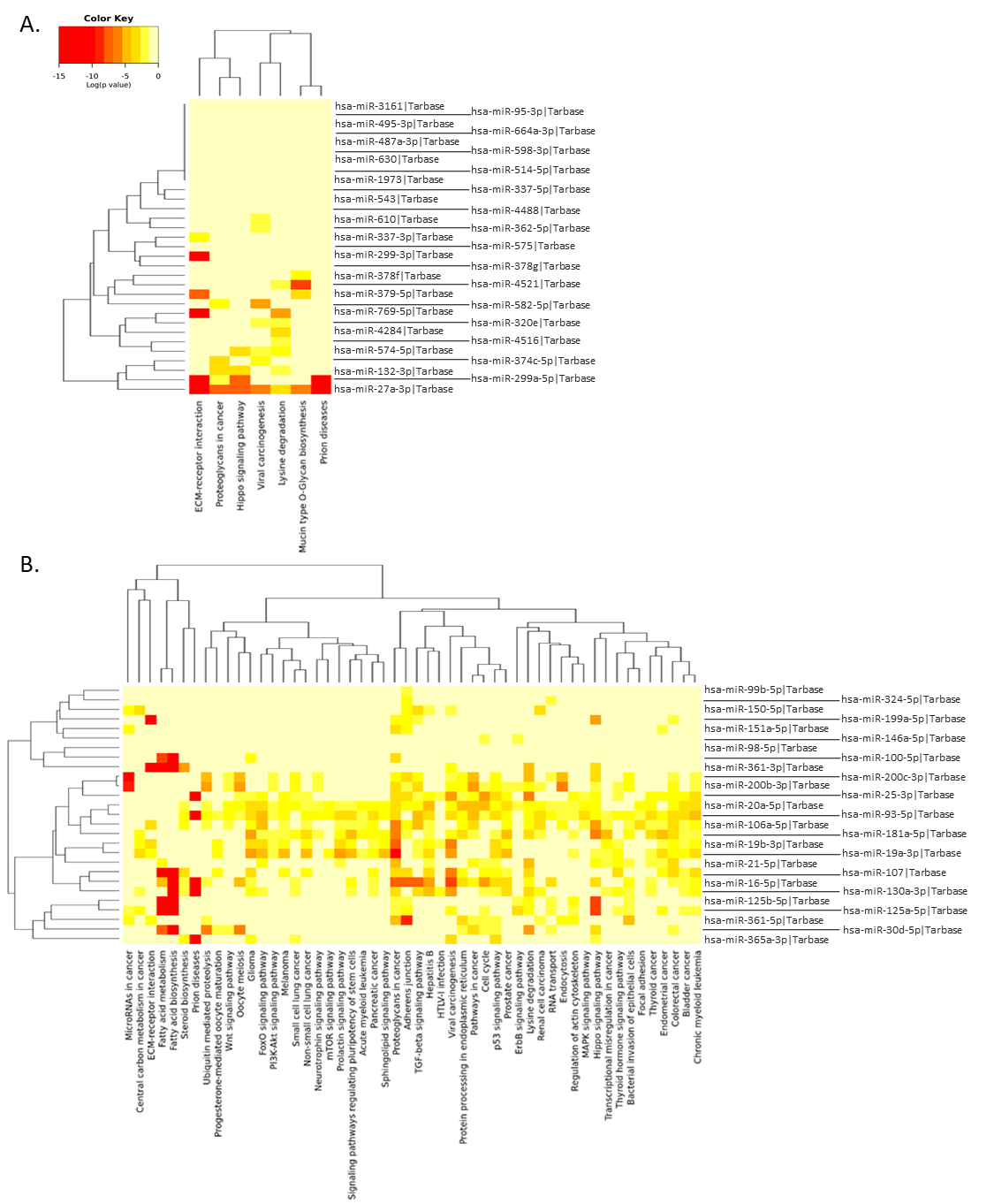
**

**Supplementary Figure 2: Ingenuity pathway analysis (IPA) of miRNAs differentially expressed between primary breast cancers that did not recur (BCNR) and the ones that recurred (BCR).** The 58 miRNAs were analysed using IPA **(A)** a number of genes, directly and/or indirectly regulated by these miRNAs, were identified. Many of these genes, such as TP53, MET, CDK4, CDKN2A, CCND3, RB, EZH2, SMAD7 and BCL6, play a significant role in **(B)** the proliferation of immune cells and the processes of **(C)** migration and **(D)** metastasis.


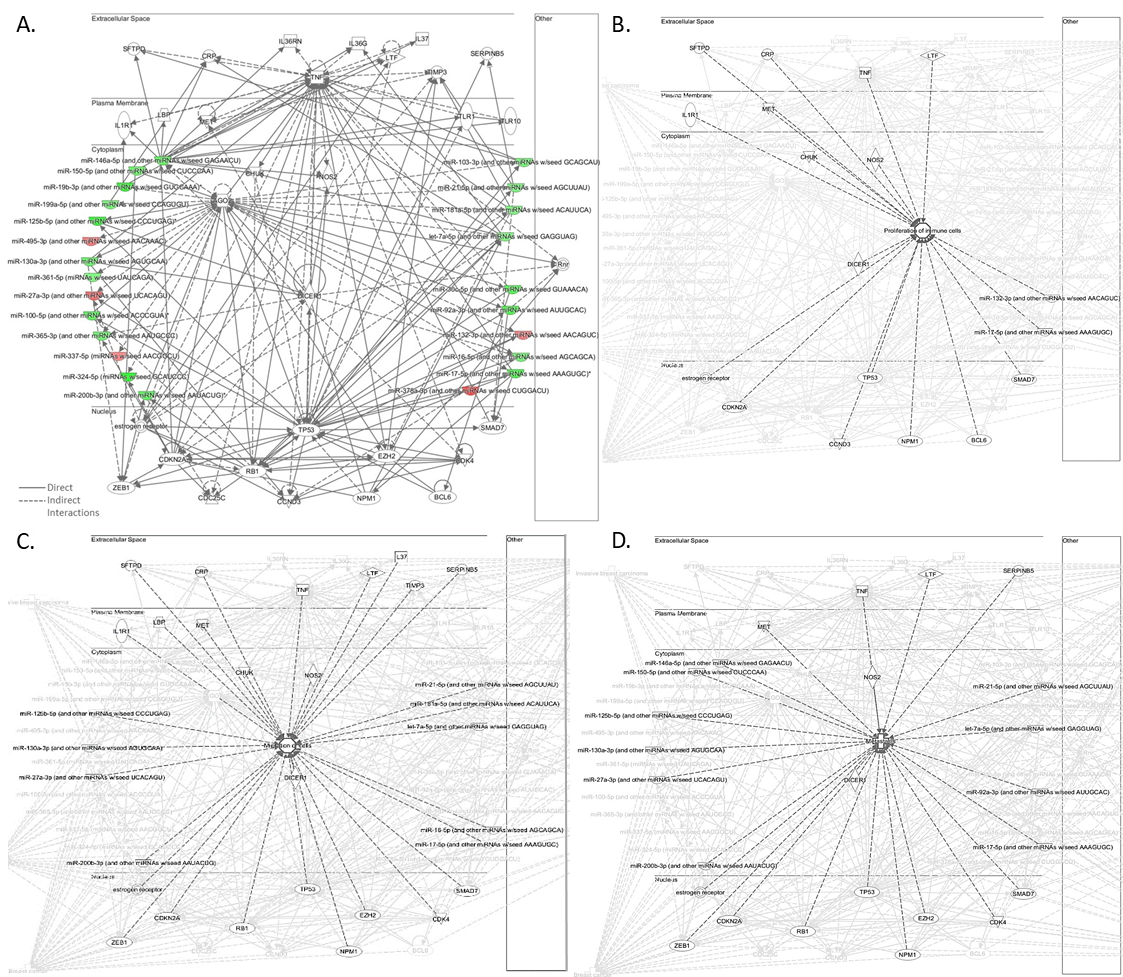


**Supplementary Figure 3: miRNA pathway enrichment analysis of primary breast cancer (BC) that recurs to the brain (BCR) and their paired breast cancer brain metastasis (BCBM).** Pathway Union heatmap of the **(A)** 2 downregulated and **(B)** 9 upregulated miRNAs in BCRs in comparison to their matched BCBMs (dark and light grey respectively in supplementary table 3). The top significant KEGG pathways are indicated. The significance of enrichment is indicated by the strength of the colour (log_10_ p value ranging from 0 to 10^-15^).


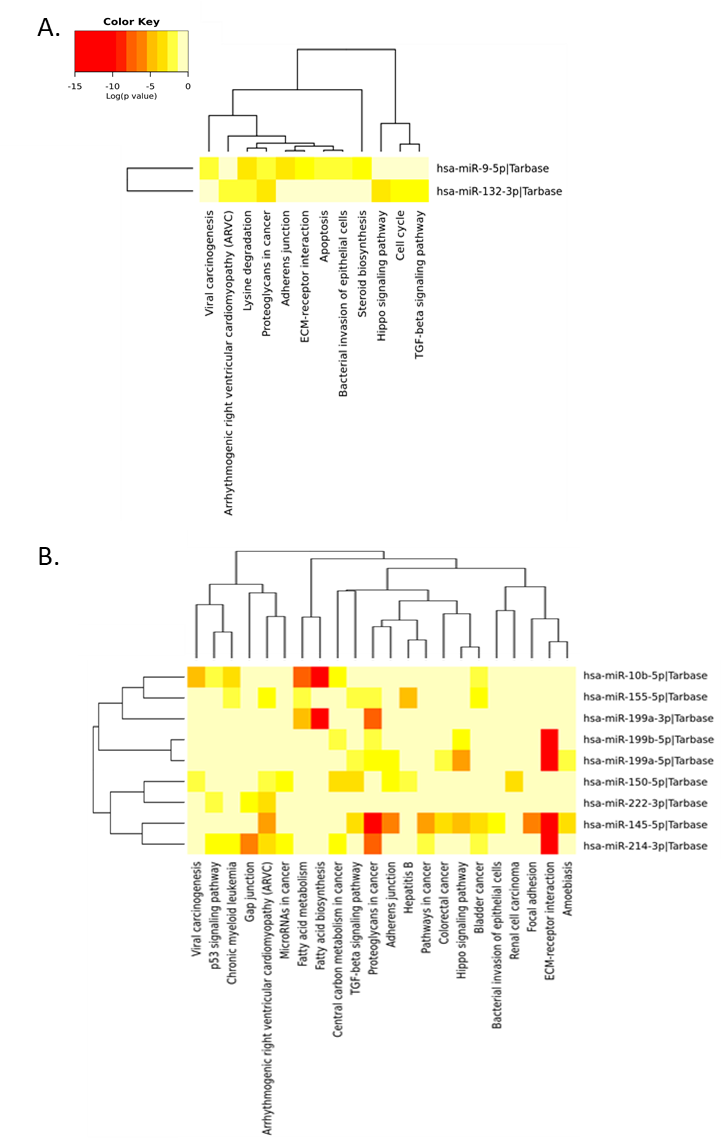


**Supplementary Figure 4: Ingenuity pathway analysis (IPA) of miRNAs differentially expressed between primary breast cancers that did recur (BCR) and their breast cancer brain metastasis (BCBM).** The 11 miRNAs were analysed using IPA **(A)** a number of genes, directly and/or indirectly regulated by these miRNAs, were identified. Many of these genes play a significant role in **(B)** the proliferation of immune cells, **(C)** the processes metastasis and **(D)** colony formation such as MET, MYC, CD44, CDH1, MUC1, TGFB1, KLF4, TNF, BIRC5, TAGLN, CDK4, TWIST1, EP300, DNMT3A, CXCL2, CXCL3, CD209.


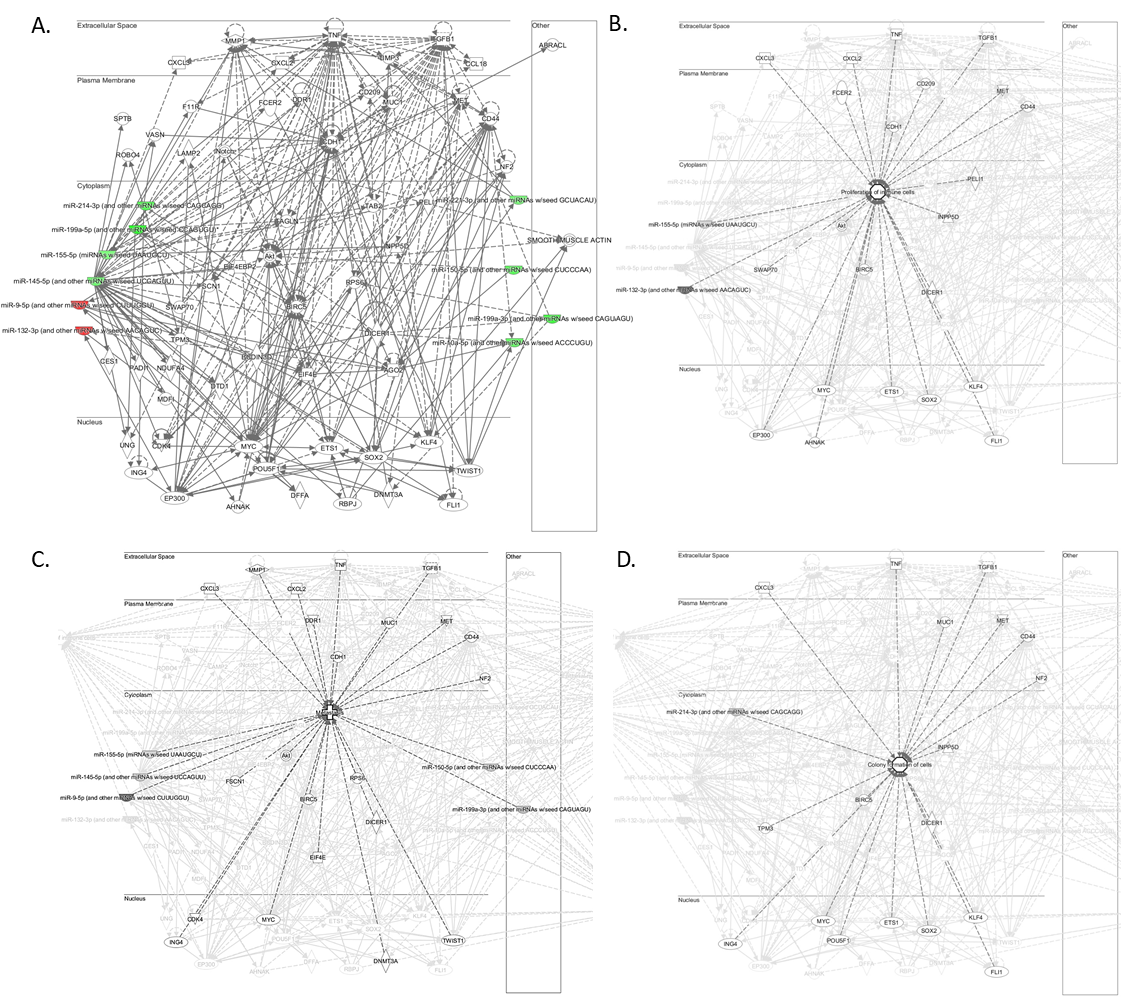


**Supplementary Figure 5: Kaplan-Meier survival analysis of the individual miRNAs.** The individual miRNA contribution in (A) brain metastasis-free survival (BMFS) and (B) overall survival (OS) is illustrated below. High miR-132-3p expression confers a protective effect whereas high miR-150-5p, miR-155-5p and miR-199a-5p confer a risky effect (opposite direction of HR). The most impactful miRNA in both BMFS and OS is the miR-132-3p, followed by miR-150-5p and miR-155-5p (risky effect). The miR-199a-5p is not significant as the arms overlap for the first 4 years (48 months). Still, it is indicated that it could be a late survival marker.


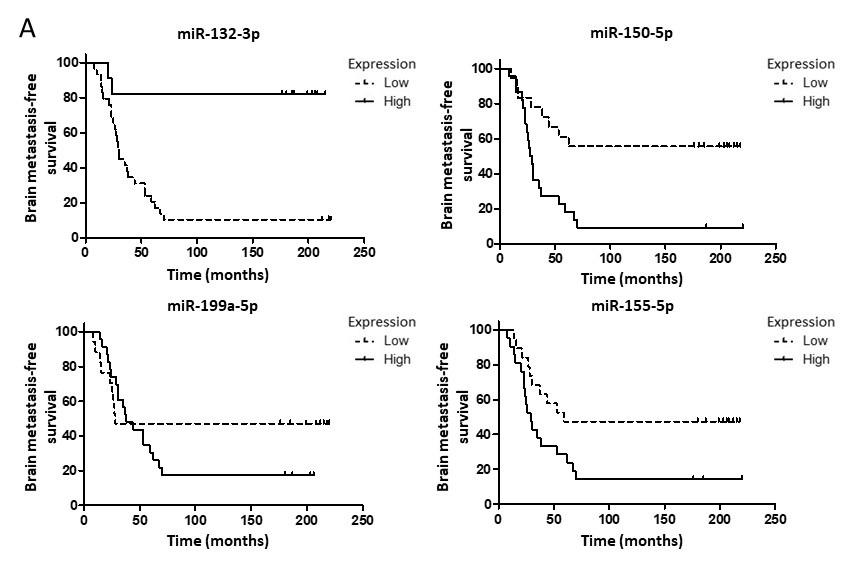


miR-132-3p: p=0.0003, HR: 0.236, 95% CI: 0.108-0.516 miR-150-5p: p=0.0019, HR: 3.378, 95% CI: 1.567-7.299

miR-155-5p: p=0.0261, HR: 2.358, 95% CI: 1.107-5.025 miR-199-5p: p=0.318, HR: 1.475, 95% CI: 0.687-3.165


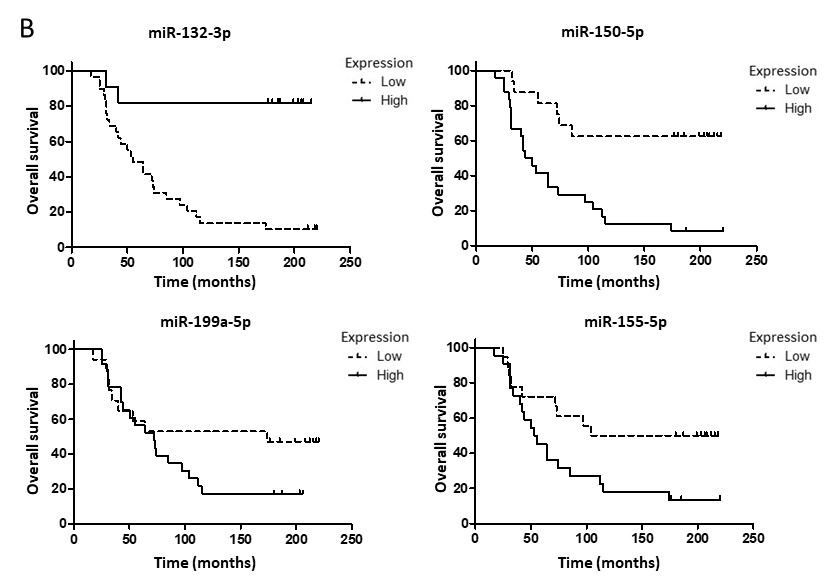


miR-132-3p: p=0.0003, HR: 0.232, 95% CI: 0.106-0.509 miR-150-5p: p=0.0004, HR: 3.922, 95% CI: 1.832-8.403

miR-155-5p: p=0.0277, HR: 2.336, 95% CI: 1.098-4.975 miR-199-5p: p=0.138, HR: 1.782, 95% CI: 0.833-3.788

**Supplementary Figure 6: Pathway enrichment analysis of the differentially expressed miRNAs between primary breast cancer that did not recur (BCNR), primary BCR and their paired breast cancer brain metastasis (BCBM).** MiRNA target enrichment canonical pathway analysis in IPA [Benjamini-Hochberg (B-H) multiple testing correction (corrected p <0.1, FDR 10%: light-grey line threshold)] linked them to pathways involved in immunity/ inflammation and molecular mechanisms of cancer. (A) Pathways identified in BCNR versus BCR: interleukin (IL-6, IL-10, IL-7, IL-8), TLR, Th1/Th2 and neuroinflammation signalling pathways. (B). Pathways identified in BCR versus BCBM: interleukin (IL-7, IL-8), PI3K signalling in B lymphocytes and neuroinflammation signalling pathways. The grey line indicates the ratio of genes from our dataset that maps to the pathway divided by the total number of genes that map to the same pathway.


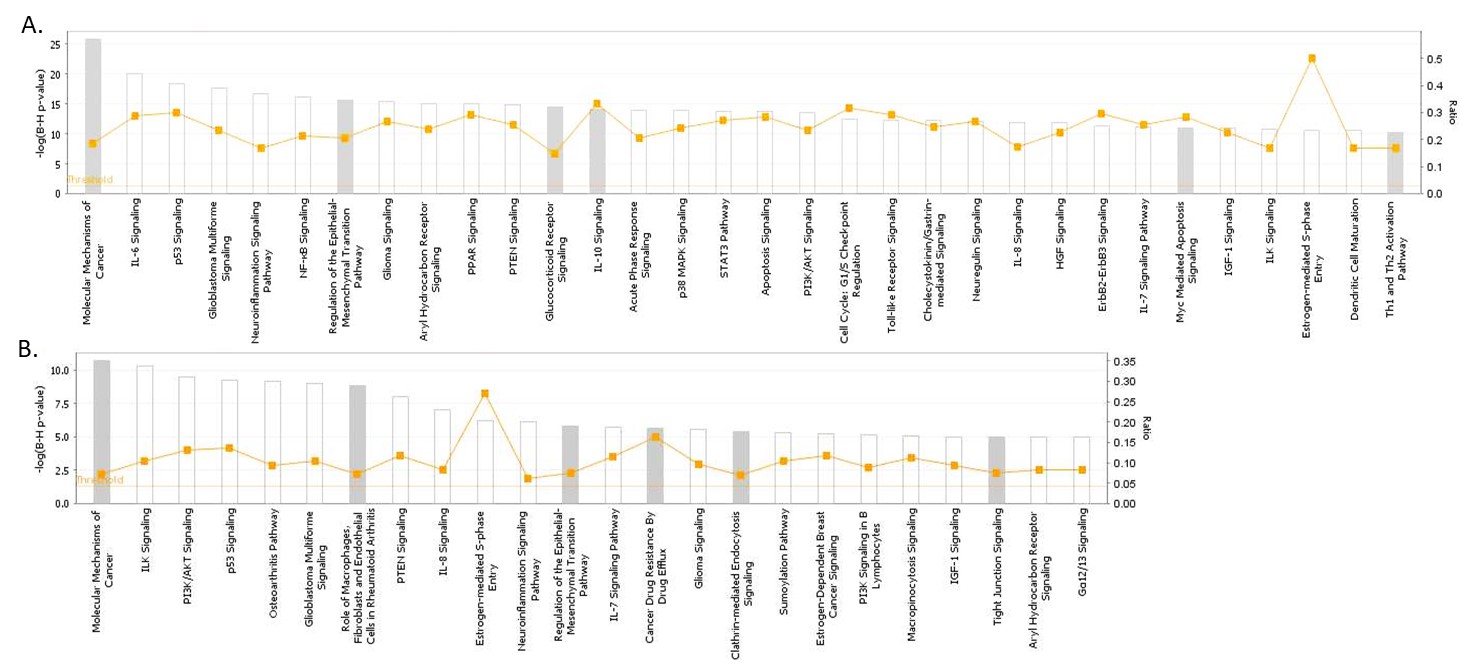

Supplement: Supplementary file 1 — SREP-19-21066A_Supplementary Info [file 41598_2019_55084_MOESM1_ESM.docx]
